# Benchmarking retrieval-augmented large language models in biomedical NLP: Application, robustness, and self-awareness

**DOI:** 10.1126/sciadv.adr1443

**Published:** 2025-11-21

**Authors:** Mingchen Li, Zaifu Zhan, Han Yang, Yongkang Xiao, Huixue Zhou, Jiatan Huang, Rui Zhang

**Affiliations:** ^1^ Division of Computational Health Sciences, Department of Surgery University of Minnesota, Minneapolis, MN, USA.; ^2^ Department of Electrical and Computer Engineering, University of Minnesota, Minneapolis, MN, USA.; ^3^ Institute for Health Informatics University of Minnesota, Minneapolis, MN, USA.

## Abstract

To reduce hallucinations in large language models (LLMs), retrieval-augmented LLMs (RALs) retrieve supporting knowledge from external databases. However, their performance on biomedical natural language processing (NLP) tasks remains underexplored. We introduce Biomedical Retrieval-Augmented Generation Benchmark, a comprehensive evaluation framework assessing RALs across five biomedical NLP tasks and 11 datasets, using four testbeds: unlabeled robustness, counterfactual robustness, diverse robustness, and self-awareness. To improve RALs’ robustness and negative awareness, we propose a detect-and-correct strategy and a contrastive learning approach. Experimental results show that RALs generally outperform standard LLMs on most biomedical tasks, but still struggle with robustness and self-awareness, particularly under counterfactual and diverse scenarios. Our proposed methods significantly improve performance in robustness to unlabeled and counterfactual data, and increase the model’s ability to detect and avoid incorrect predictions. These findings highlight key limitations in current RALs and underscore the need for continued refinement to ensure reliability and accuracy in high-stakes biomedical applications.

## Teaser

BioRAB evaluates retrieval-augmented language models on robustness and self-awareness in biomedical NLP tasks.

## INTRODUCTION

Recently, substantial progress has been made in large language models (LLMs). To adapt the LLM to the biomedical domain, several biomedical focused LLMs have been developed, such as MedLLaMA-13B (*1*) and Med-PaLM 2 (*2*). Despite demonstrating impressive general capabilities (*3*), these models still face substantial challenges, including factual hallucination (*4*) and absence of newly uploaded knowledge (*5*).

Retrieval-augmented language models (RALs) (*3*, *6*–*8*), in contrast, can retrieve knowledge from an external datastore when needed, potentially reducing hallucination and improving the new knowledge adaption ability. The most common method is to use the designed retriever to retrieve the knowledge that is relevant to the input sentence; subsequently, the retrieved knowledge, along with the input sentence, is fed into the LLM to assist in generating the expected output.

In the question answering (QA) task, a RAL can access knowledge from an unlabeled corpus, which serves as a retrievable knowledge base, such as PubMed. The QA format allows the unlabeled corpus to potentially furnish answers to questions. However, for tasks like triple extraction, incorporating the unlabeled corpus may yield adverse effects. Counterfactual information, such as error annotations, is prevalent in labeled corpora, presenting challenges for retrievers in obtaining useful information. In addition, LLMs still grapple with generating unreliable information retrieved incorrectly. Incorporating diverse knowledge holds promise for improving model performance. For example, QA relies on extracting information from extensive contexts, thus potentially affecting information extraction performance. Moreover, the influence of retrieval information from various tasks or datasets on RAL performance remains underexplored. Self-awareness is crucial for RALs; if RALs can distinguish between positive retrieval knowledge and negative knowledge, they have the opportunity to rectify their actions.

**Fig. 1.**
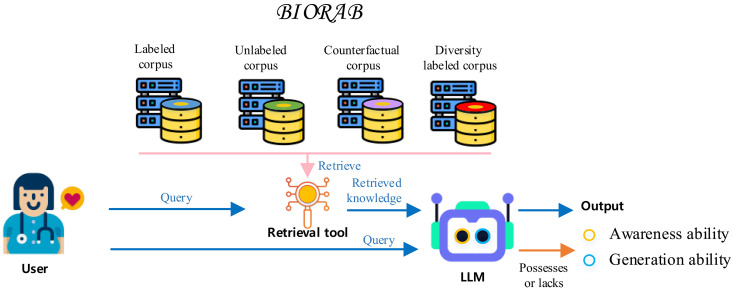
BioRAB features on queries on different types of corpus to test the awareness ability and generation ability of RAL.

These challenges hinder RALs from consistently producing reliable and accurate responses. Unfortunately, in the biomedical domain, only a few studies, such as Almanac (*9*), have explored the RAL performance in QA, leaving a gap in understanding how these factors affect RAL across various biomedical natural language processing (NLP) tasks. Consequently, there is a pressing need for a comprehensive evaluation of RALs with different LLMs across biomedical NLP tasks. To this end, this paper conducts a comprehensive evaluation of RAL for different LLMs on five biomedical NLP tasks over 11 datasets. Specifically, we create a new RAL benchmark for biomedical NLP, namely, Biomedical Retrieval-Augmented Generation Benchmark (BioRAB), as shown in Fig. 1, and create four testbeds to evaluate the mentioned fundamental abilities.

1. Unlabeled robustness denotes the ability of RALs to extract valuable information from unlabeled retrieval corpus, especially on label-intensive tasks, such as triple extraction and classification. For instance, in tasks like relation extraction, the corpus could be a labeled dataset (such as the training dataset) or unlabeled (training dataset without labels). If the RAL achieves comparable or superior performance by retrieving the unlabeled dataset compared to retrieving the labeled dataset, it indicates that labeled databases may not be necessary for RALs. In the testbed of the unlabeled robustness, the corpus contains instances without labels.

2. Counterfactual robustness denotes whether the RAL could retrieve the right information from the counterfactual corpus; in our work, the counterfactual instance refers to the mislabeled annotation. In the testbed of counterfactual robustness, the corpus consists of instances with a certain proportion of incorrect labels.

3. Diverse robustness evaluates whether RALs can achieve better performance by integrating information from multiple tasks. For instance, the corpus for the classification task is sourced from relation extraction and QA tasks. In the testbed of diverse robustness, the corpus comprises instances from various tasks.

4. Negative awareness refers to the RAL’s ability to discern whether retrieved knowledge positively or negatively affects the final output. In the testbed of negative awareness, the corpus comprises instances that are 100% counterfactual instances.

Using BioRAB, we evaluate its performance across five tasks (triple extraction, link prediction, text classification, QA, and natural language inference) using 11 biomedical NLP datasets. Furthermore, BioRAB undergoes evaluation with five widely used LLMs: LLaMA2-13B (*10*), MedLLaMA-13B (*1*), LLaMA3-8B (*11*), Phi4 14B (*12*), and Qwen2.5 32B (*13*), using three commonly used retrievers [BM25 (*14*), Contriever (*15*), and MedCPT (*16*)].

We observed that although RALs can enhance response accuracy in the majority of biomedical NLP tasks we evaluated, they encounter notable challenges. Particularly in the QA task, we noted that RALs did not yield significant improvements in the datasets we used. We speculate that this could be attributed to the limitations of the corpus used for retrieving, as the training dataset (corpus we used for retrieving) may not have provided sufficient information compared to using Wikipedia or PubMed. Moreover, RALs struggle to generate the desired output when the corpus lacks labeling when compared to the labeled corpus. An interesting finding is that in datasets like ChemProt and Hetionet, RALs exhibit improved performance with unlabeled corpora compared to the source LLM. Besides, RALs lack the capability to extract useful information from counterfactual corpora and struggle to discern the most relevant information. We also find that this is not a common case; some datasets, such as in the dataset ADE and Hetionet, RAL could handle the counterfactual instance. In addition, when presented with a diverse labeled corpus, RALs do not achieve optimal performance across tasks, except for the natural language inference task. Last, we found that despite providing counterfactual examples during training, the LLM was still able to generate correct outputs in some instances. However, RALs struggle with self-awareness, as they lack the ability to determine which examples could help improve model performance.

To address these challenges, we propose a detect-and-correct method aimed at enhancing RAL’s ability to identify and rectify errors in the retrieved corpus, thereby improving its overall reliability. In addition, we introduce a contrastive learning approach that refines RAL’s negative awareness by strengthening its capacity to distinguish differences of samples. By integrating these two techniques, our method significantly enhances RAL’s robustness and awareness ability, making it more effective in processing complex retrieval tasks. Together, these improvements contribute to a more resilient and accurate model for biomedical applications.

Our contributions are the following:

1. We propose four abilities essential for evaluating RALs in the biomedical domain and introduce a benchmark called BioRAB to assess these capabilities. To our knowledge, this is the first benchmark tailored specifically to evaluate these four abilities for RALs in the biomedical domain.

2. We propose a detect-and-correct method and a contrastive learning approach to enhance the RAL’s robustness (unlabeled and counterfactual) and negative awareness.

3. We evaluated the LLM using the retrieval-augmented method and identified limitations in four key abilities.

4. We evaluate the RAL on five different biomedical tasks over 11 datasets by using five LLMs with three retrievers.

## RESULTS

### Results of RALs and backbone LLMs

We first benchmark various LLMs and RALs on 11 datasets; the results are shown in Table 1. In the triple extraction task, we observed that RALs outperformed LLMs (specifically RALs without a retriever) on LLaMA2-13B, MedLLaMA-13B, LLaMA3-8B, and phi4 14B, achieving better performance. For example, RALs (MedLLaMA 13B with Contriever) enhanced the original MedLLaMA 13B by 22.37%, in terms of F1 score on the ADE dataset. Another finding is that for Qwen2.5-32B, using different retrievers does not lead to significant performance improvement.

**Table 1.** Micro F1 results for triple extraction, link prediction, and text classification, along with macro-average F1 for question answering and BioNLI, across various approaches on 11 datasets. Underline indicates the best performance on each dataset. For complete results, please refer to the Supplementary Materials.

		Triple extraction			Link prediction			Classification			QA	NL inference
LLM	Approach	ADE	ChemProt	GIT	PHarmKG	Hetionet	DS	Ade-corpus-v2	SemClass	SDoH classification	MedMCQA	BioNLI
LLaMA2-13B	BM25 (*14*)	30.93	49.11	57.61	97.60	82.37	75.86	95.40	75.50	63.71	40.42	45.10
	Contriever (*15*)	36.06	85.40	73.55	98.00	77.00	76.50	96.60	79.33	61.18	35.52	35.12
	MedCPT (*16*)	30.81	85.82	74.52	97.40	81.60	78.01	96.80	78.33	68.77	36.80	69.21
	No Retriever	34.86	77.48	58.99	97.60	80.80	77.15	96.40	77.66	72.57	41.52	62.62
MedLLaMA-13B	BM25 (*14*)	33.77	52.02	57.89	95.00	90.04	71.33	95.60	72.67	65.40	37.86	48.81
	Contriever (*15*)	34.58	85.69	65.88	97.00	77.20	75.64	95.60	77.66	56.96	29.77	53.07
	MedCPT (*16*)	31.41	80.70	75.24	97.40	84.40	72.84	95.40	76.16	66.66	33.88	53.68
	No Retriever	12.21	50.52	42.05	97.20	78.54	80.38	95.40	64.00	67.71	46.47	61.07
LLaMA3-8B	BM25 (*14*)	27.79	70.10	62.65	96.80	81.80	75.43	94.80	75.50	71.30	37.79	19.17
	Contriever (*15*)	32.70	86.91	70.96	96.60	73.40	78.01	94.60	75.83	72.57	28.11	63.85
	MedCPT (*16*)	29.19	84.81	57.59	97.00	83.00	76.29	95.40	74.67	71.72	31.56	56.89
	No Retriever	7.05	21.32	74.27	97.20	81.80	80.38	93.80	73.16	57.00	55.91	6.71
Phi4 14B	BM25 (*14*)	36.23	81.26	60.61	97.20	88.80	60.75	88.20	74.67	78.90	35.49	67.65
	Contriever (*15*)	27.66	69.70	74.84	31.60	74.20	53.58	90.80	75.17	81.43	33.89	56.65
	MedCPT (*16*)	26.35	79.48	72.55	96.80	82.80	57.38	90.60	75.00	80.16	27.72	85.45
	No Retriever	16.56	77.23	30.09	97.60	82.20	54.05	89.20	72.50	74.68	66.18	82.50
Qwen2.5 32B	BM25 (*14*)	40.56	86.24	65.51	97.20	82.00	72.44	94.20	78.50	78.05	28.39	81.29
	Contriever (*15*)	45.65	87.16	73.12	97.60	75.80	77.37	94.20	76.33	80.16	28.10	89.99
	MedCPT (*16*)	42.62	87.72	69.46	96.20	82.80	71.78	95.00	76.83	72.99	28.22	91.19
	No Retriever	26.76	88.09	68.50	90.40	81.00	76.93	94.80	48.00	74.26	20.91	63.20

RALs have also been evaluated as effective in improving the performance of LLMs across tasks such as link prediction, text classification, and natural language inference. RALs (LLaMA2 13B with Contriever) enhanced the original LLaMA2 13B by 0.40%, in terms of F1 score on the PHarmKG dataset; RALs (MedLLaMA 13B with BM25) enhanced the original MedLLaMA 13B by 11.86%, in terms of F1 score on the Hetionet dataset; RALs (LLaMA2 13B with MedCPT) enhanced the original LLaMA2 13B by 0.40%, in terms of F1 score on the Ade-corpus-v2 dataset; RALs (LLaMA2 13B with Contriever) enhanced the original LLaMA2 13B by 1.67%, in terms of F1 score on the SemClass dataset; RALs (LLaMA2 13B with MedCPT) enhanced the original LLaMA2 13B by 6.59%, in terms of Macro-avg F1 on the BioNLI dataset.

On MedMCQA, our findings differ from other works (*17*) as we observed that LLMs outperform RALs in achieving the best performance, and we speculate that the reason for this discrepancy lies in the nature of label-insensitive model tasks, where RALs have the capability to retrieve large corpora such as PubMed (*18*) or other relevant datasets. In our study, however, our corpus is derived solely from the training set, which may limit the breadth of knowledge accessible to the RALs.

### Results of testbeds 1, 2 and 3

We evaluate the model performance based on the unlabeled corpus, and the results are shown in Table 2. We have the following observations:

**Table 2.** Micro F1 results for triple extraction, link prediction, text classification, along with macro-average F1 for BioNLI, across various approaches on EIGHT datasets under testbed 1, testbed 2, and testbed 3. Underline indicates the best performance on each dataset. Note: we use the best RAL in Table 1 as the backbone.

	Triple extraction		Link prediction		Classification			NL Inference
Corpus	ADE	GIT	PHarmKG	Hetionet	Ade-corpus-v2	SemClass	SDoH classification	BioNLI
Unlabeled corpus	14.39	1.01	97.20	78.60	93.00	6.83	72.99	61.38
Counterfactual corpus (20%)	45.06	73.03	97.40	94.80	95.80	73.33	75.50	87.62
Counterfactual corpus (80%)	30.07	74.84	97.80	85.26	95.00	75.66	13.34	60.09
Counterfactual corpus (100%)	39.98	74.81	97.60	76.60	96.80	77.66	0	58.52
Diverse corpus	10.08	69.80	97.20	75.41	96.20	75.33	83.90	80.32
Labeled corpus	45.65	75.24	98.00	90.40	96.80	79.33	81.43	91.19
None	26.76	42.05	97.60	78.54	96.40	77.66	74.68	63.20

1. RAL using the unlabeled corpus exhibits lower performance compared to RAL using the labeled corpus. RALs have demonstrated a strong dependence on the labeled corpus, especially on the label-intensive tasks. For instance, with labeled corpus, the performance of RAL surpasses that of RAL without labeled corpus by 26.41% on ADE.

2. Even without an unlabeled corpus, RAL still contributes to improving LLM performance in certain tasks. As shown in Table 2, on Chemprot and Hetionet, RAL using an unlabeled corpus could enhance the original LLM’s performance by 30.16 and 0.06%, respectively. We speculate that LLMs may have sufficient knowledge to contribute to enhancing model performance on specific datasets.

We evaluate the model performance based on different counterfactual rates, and the results are shown in Table 2. We have the following observations:

1. Counterfactual corpus has a challenge for RALs. On ADE, counterfactual instances significantly influence the model performance. For instance, when the counterfactual rate is set to 80%, the triple F1 drops to around 10%, showcasing a considerable disparity compared to the triple F1 performance on the labeled corpus. Similar observations are noted in GIT, PharmKG, Ade-corpus-v2, SemClass, and BioNLI. This suggests that RALs can be easily misled by counterfactual corpus.

2. A lower counterfactual rate may have a reduced impact on RALs. On Hetionet, we observed that when the counterfactual corpus is set to 20%, the model performance is better than the factual corpus. We speculate that retrievers have a greater chance of obtaining useful information when the counterfactual rate is lower.

3. The counterfactual corpus can still contribute to improving LLM performance. On ADE, GIT, PHarmKG, Hetionet, Ade-corpus-v2, SemClass, and BIoNLI, the interesting finding is that even with a counterfactual corpus, the RAL performance often surpasses the original LLM. We speculate that the counterfactual corpus may have a beneficial effect on LLMs. Despite the content of the instances being counterfactual, the provided templates still aid in generation.

4. Counterfactual rates and model performance are not inversely proportional. This finding contradicts human intuition. In some datasets, such as SemClass, when the counterfactual rate is higher, the model performance also improves. This suggests that RALs have a certain ability to handle counterfactual facts.

We evaluate the model performance of diversity robustness, and the results are shown in Table 2. We have the following observations:

The diversity-labeled corpus poses a challenge to improve RALs. We found that RALs consider the knowledge in the diverse corpus as noise, which could potentially affect RAL performance, particularly evident in ADE datasets. However, on BioNLI, the diversity-labeled corpus could contribute to enhancing the model performance. We speculate that one reason is the retriever we used could not retrieve useful information, while another reason could be that the corpus lacks the necessary information.

In addition to the public dataset, we also used our labeled private dataset, Social Determinants of Health (SDoH) Classification, which is derived from real-world data. We observed the same conclusion as with the public dataset: The unlabeled dataset and counterfactual corpus influence the performance of RALs. We did not include our labeled private dataset DS, as RALs without retrievers achieved better performance.

### Results of testbed 4: Negative awareness

We evaluate the model performance of negative awareness, and the results are shown in Table 3. We have the following observations:

**Table 3.** RAL performance of ADE, GIT, PHarmKG, Hetionet, Ade-corpus-V2, SemClass, SDoH, and BIoNLI on testbed 4: negative awareness.

Task	Dataset	True negative awareness rate	Fake negative awareness rate
Triple extraction	ADE	5.75	94.24
	GIT	27.73	69.75
Link prediction	PHarmKG	0.00	63.11
	Hetionet	1.71	31.33
Text classification	Ade-corpus-v2	68.75	70.45
	SemClass	1.49	99.35
	SDoH classification	11.18	86.22
Natural language inference	BioNLI	0.00	0.38

RAL poses a challenge to the negative awareness. The true negative awareness rate on PharmKG and BioNLI was zero, and it was only 1.07% on ADE. The overall performance of fake negative awareness is better than that of true negative awareness. This suggests that RALs still struggle with self-awareness regarding which examples could provide useful information for generations.

### Results of our method on the unlabeled database

Table 4 presents the results of our detect-and-correct method compared to the RAL with different retrieval corpus settings for text classification on Ade-corpus-v2 and SemClass. Our method achieves an F1 score of 94.00% on Ade-corpus-v2 and 75.17% on SemClass, showing competitive performance. The RAL with labeled corpus yields the highest scores, with 96.80 and 79.33% F1 scores, respectively, while the RAL with unlabeled corpus performs poorly on SemClass (6.83% F1 score). Notably, our detect-and-correct method outperforms the RAL with an unlabeled corpus, demonstrating its effectiveness in improving robustness. These results highlight the impact of correction-based approaches in text classification tasks. Note: To ensure fairness, we use the best-performing RAL from Table 3 as the backbone for each task.

**Table 4.** Results of our detect-and-correct method on the text classification task demonstrate improved robustness when using an unlabeled corpus in the retrieval progress. Note: we use the same RAL for different corpus.

	Ade-corpus-v2			SemClass		
Corpus	Precision	Recall	F1	Precision	Recall	F1
Unlabeled corpus	93.00	93.00	93.00	6.83	6.83	6.83
Labeled corpus	96.80	96.80	96.80	79.33	79.33	79.33
None	96.40	96.40	96.40	77.66	77.66	77.66
Detect-and-correct	94.00	94.00	94.00	75.17	75.17	75.17

### Results of our method on the counterfactual database

Table 5 reports the F1 scores of RAL under five corpus settings across two biomedical benchmarks: PharmKG and BioNLI. Our proposed detect-and-correct framework enhances retrieval by leveraging GPT4 to revise retrieved counterfactual samples before model inference. This method substantially improves performance on BioNLI, achieving an F1 score of 72.77%, outperforming all other retrieval configurations. On PharmKG, although the RAL with labeled corpus yields the highest F1 score (98.00%), our method performs comparably (97.80%), demonstrating the efficacy of counterfactual correction in mitigating retrieval noise.

**Table 5.** Results of our detect-and-correct method on the PharmKG and BioNLI.

	PharmKG	BioNLI
Corpus	F1	F1
Counterfactual corpus (100%)	97.60	58.52
Unlabeled corpus	78.60	61.38
Labeled corpus	98.00	91.19
None	97.60	63.20
Detect-and-correct	97.80	72.77

### Results of our method on awareness

By calculating the true negative awareness rate and fake negative awareness rate of the best RALs on the SDoH classification, we found that our method, which pretrains the model using RAL to improve its ability to distinguish between positive and negative instances, achieves a 49.56% awareness rate, compared to 48.70% for RAL alone. These results indicate that our approach enhances the model’s ability to differentiate between classes. The improvement suggests that incorporating distinguishing mechanisms strengthens classification robustness.

### Sampling bias

To investigate how sampling bias affects evaluation, we conducted simulation studies on a two-label classification task. We constructed datasets with different positive-to-negative ratios of 1:1 and 1:5 and reported the results for weighted precision, recall, F1 score, AUROC (area under the receiver operating characteristic curve), and AUPRC (area under the precision–recall curve). In these experiments, we used the best-performing RAL LLaMA2-13B + MedCPT for Ade-corpus-v2 and LLaMA2-13B + Contriever for SemClass, respectively. Table 6 presents performance metrics across different datasets (Ade-corpus-v2 and SemClass) with varying positive-negative ratios (1:1 versus 1:5). The reported metrics include Micro Precision, Micro Recall, Micro F1 score, Weighted Precision, Weighted Recall, Weighted F1 score, AUROC, and AUPRC.

**Table 6.** Performance metrics for different datasets and positive-negative ratios.

Dataset	Positive/ Negative ratio	Micro precision	Micro recall	Micro F1	Weighted precision	Weighted recall	Weighted F1	AUROC	AUPRC
Ade-corpus-v2	1:1	93.40	93.40	93.40	93.39	93.40	93.52	93.91	90.23
Ade-corpus-v2	1:5	93.60	93.60	93.60	93.66	93.60	93.63	92.58	90.38
SemClass	1:1	66.50	66.50	66.50	68.86	66.50	66.08	67.40	77.47
SemClass	1:5	48.50	48.50	48.50	63.38	48.50	35.04	51.75	66.62

For Ade-corpus-v2, the model performs consistently across both balanced (1:1) and imbalanced (1:5) datasets, with minimal variation in F1 score and AUROC. For SemClass, there is a significant drop in performance when shifting from a balanced 1:1 ratio (F1 score: 66.50%, AUROC: 67.40%) to an imbalanced 1:5 ratio (F1 score: 48.50%, AUROC: 51.75%). This suggests that class imbalance significantly affects performance in SemClass, leading to reduced classification robustness. 

The degradation in SemClass performance under imbalance indicates that the model struggles to generalize when one class is underrepresented in training data. While AUROC remains high in Ade-corpus-v2, the SemClass model under 1:5 imbalance sees a drastic drop in AUROC (67.40% → 51.75%), highlighting the difficulty in distinguishing between positive and negative samples. A similar trend is observed in Weighted F1 score, which drops from 66.08 (1:1) to 35.04 (1:5), showing that overrepresenting the negative class leads to biased predictions.

### Error analysis

#### Testbed 1

In this part, we use ADE, GIT, and BioNLI as examples for error analysis. To better understand the impact of the unlabeled corpus on model generation, this part primarily analyzes the RAL performance on ADE, GIT, and BioNLI, which exhibited the poorest performance among the nine datasets used. We primarily summarize two error types as shown in Table 7. We observed that with the unlabeled corpus, RAL tends to generate redundant information and struggles to accurately predict the output, such as the head entity or relation type in the triple extraction task.

**Table 7.** Error cases of unlabeled robustness. In BioNLI, we have not included the input sentence in this table due to the excessive length of the sentences.

Error type	Dataset	Input sentence	Expected output	Error output
Redundant information	ADE	The fourth patient showed rls symptoms that were initially caused by a 20-mg daily olanzapine dosage and were later mitigated when olanzapine was reduced and ropinirole was administered.	{Olanzapine, dosage, 20 mg daily}	{Olanzapine, dosage, rls symptoms that were initially caused by a 20-mg dail}
	GIT	Inactivation kinetics of bacterial glycerol dehydratase (ec 4.2.1.30) in the course of its reaction with adenosylcobalamin (adocbl) and its analogs were investigated.	Glycerol dehydratase	Adenosylcobalamin.. retrieved sentence: glycerol dehydratase
	BIONLI	–	Negative	Negative retrieved sentence..
Error generation	ADE	Four patients receiving high-dose tamoxifen for greater than 1 year have demonstrated similar retinal changes.	(Tamoxifen, dosage, high-dose)	(Tamoxifen, effect, retinal changes.)
	GIT	Inactivation of serum alkaline phosphatase by adrenaline and related substances	(Adrenaline, inhibits, alkaline phosphatase)	(Alkaline phosphatase, interacts with, adrenaline)
	BIONLI	–	Positive	Negative

#### Testbed 2

In this part, we use PharmKG and SemClass as examples for error analysis. For the query “What is the relationship between ethanol and GABRA1?” in PharmKG, the ground truth label is “Interactions.” However, when the retrieved corpus contains 80% noise, the generated answer is “Chemical-Gene”, indicating that excessive noise in retrieval can mislead the model and result in incorrect predictions. For example in the SemClass, the model is asked to determine whether the triple (nitrous acid, INHIBITS, protein) is correctly inferred from the sentence “Inactivation of protein in poliovirus by nitrous acid.” The ground truth label is True, indicating that the relationship exists. However, the model predicts False, suggesting it fails to recognize the inhibitory relationship expressed in the sentence. This misclassification may stem from the model struggling with implicit linguistic cues, misunderstanding passive voice structures, or failing to generalize inhibition-related terms effectively.

#### Testbed 3

In this part, we use ADE and MedMCQA as examples for error analysis. On ADE, we discovered that the Diversity-labeled corpus also leads to redundancy in RAL generation, for instance, in sentence “easily reversible hypoxemia and hypotension induced by nimodipine.”, the expected tail entity is “hypotension,” while RAL regarded the “hypoxemia and hypotension induced by nimodipine.” as the entity. It also struggles with extracting complex entities. For example, in the sentence “clinical, spectroscopic, and imaging abnormalities resolved with discontinuation of metronidazole,” “clinical, spectroscopic, and imaging abnormalities” is considered the ground truth, while RAL regards the entire sentence “clinical, spectroscopic, and imaging abnormalities resolved with discontinuation of metronidazole” as a single entity. In summary, we find that the primary challenge lies in entity recognition, especially in the recognition of tail entities. On MedMCQA, we observed that error generation primarily stemmed from misjudgment. For instance, in sentence “Question: All of the following muscles are elevators of the mandible EXCEPT: Options: (A) Digastric; (B) Masseter; (C) Medial pterygoid; (D) Temporalis,” the ground truth is “A,” while RAL generates the “D.”

## DISCUSSION

### Testbeds and our methods

The results demonstrate that the reliance on labeled versus unlabeled corpora significantly affects model performance, particularly in label-intensive tasks like ADE, where labeled data improve results by 26.41%. Despite this, RAL still enhances LLM performance even when relying on an unlabeled corpus, as seen in Chemprot and Hetionet, suggesting that LLMs contain useful intrinsic knowledge. Counterfactual corpora introduce challenges, with high counterfactual rates (80%) reducing F1 scores to around 10%, indicating model susceptibility to misleading information. Counterfactual corpora sometimes enhance performance, as observed in SemClass, suggesting that even incorrect instances can contribute useful templates for generation. This phenomenon implies that RALs can handle counterfactual information to some extent, contradicting initial expectations. In addition, diversity within labeled corpora poses a challenge for RALs, particularly in ADE, where the model struggles to distinguish noise from valuable knowledge. Negative awareness remains a weakness, with RAL performing poorly in distinguishing false negatives, as evident in PharmKG and BioNLI. However, our detect-and-correct method significantly improves text classification robustness, outperforming the unlabeled corpus in SemClass. These findings highlight the importance of correction-based approaches and tailored retrieval mechanisms for optimizing model performance across diverse datasets. Otherwise, by leveraging contrastive learning methods, RAL can enhance its ability to distinguish between positive and negative instances, thereby improving its awareness and overall performance.

### Future work

To improve the unlabeled robustness, we plan to explore ways to improve label generation through pretraining, fine-tuning, or retrieval-based methods to enhance the model’s labeling capability. Another promising direction is training retrieval models to quickly adapt to new label-intensive tasks with minimal supervision, thereby improving generalization in low-resource settings. These efforts will further strengthen the robustness of RAL when handling unlabeled data, making it more effective for real-world biomedical applications.

To enhance RALs’ ability to handle counterfactual corpora, we plan to explore the following directions. First, we aim to develop retrieval strategies that incorporate uncertainty estimation, allowing RALs to weigh retrieved instances based on their likelihood of being counterfactual. Second, we propose using contrastive objectives to train RALs to distinguish between factual and counterfactual instances, thereby improving retrieval robustness against misleading examples.

To enhance retrieval robustness, incorporating uncertainty-aware filtering and hybrid retrieval (dense + sparse methods) could help reduce reliance on misleading information. Addressing negative awareness requires contrastive learning for retrieval filtering and adversarial training on counterfactual data to improve the model’s ability to recognize false negatives. In addition, domain-specific adaptation can be improved by pretraining on biomedical corpora and fine-tuning with structured knowledge graphs to enhance factual consistency. Future work should explore these strategies to make RALs more reliable in complex biomedical tasks.

To quantify hallucinations, evaluation testbeds should include biomedical fact-verification benchmarks that measure accuracy in factual claims. Metrics such as retrieval grounding score can assess how well model outputs align with retrieved evidence, while hallucination rate can track the proportion of responses containing unverifiable or incorrect claims. Implementing uncertainty-aware retrieval filtering would further help reduce the inclusion of low-confidence or misleading retrievals. Future research could also explore multihop retrieval reasoning, where RALs retrieve multiple supporting documents to validate complex biomedical claims. These techniques would significantly enhance the reliability of RALs in clinical decision support, biomedical literature analysis, and drug discovery tasks.

Last, while our study advances methods for retrieval-augmented learning in biomedical domains, it does not include comparative evaluations with other advanced LLMs. Addressing this limitation in future work will be important to assess the broader applicability and generalizability of our findings.

## MATERIALS AND METHODS

In this part, we assess RAL’s performance across various biomedical NLP tasks, analyze its efficacy on four proposed testbeds, and discuss its abilities.

### Settings and dataset

We evaluated five state-of-the-art LLMs—LLamA2-13B, MedLLamA-13B, LLaMA3 8B, Phi4 14B, and Qwen2.5 32B—along with three retrievers—BM25, Contriver, and MedCPT. We considered five biomedical NLP tasks: triple extraction, link prediction, text classification, QA, and natural language inference, across 11 datasets: ADE, ChemProt, GIT, PHarmKG, Hetionet, Ade-corpus-v2, SemedCLass, MedMCQA, BioNLI, DS, and SDoH. The data statistics are shown in Table 8. The experiments were conducted using A100 GPUs.

**Table 8.** Data statistics for the datasets we used in this work.

	Dataset	Train	Test	Dev
Triple extraction	ADE (*21*)	4970	2130	–
	ChemProt (*22*)	4001	3355	2366
	GIT (*23*)	3734	465	492
Link prediction	PHarmKG (*24*)	4000	500	500
	Hetionet (*25*)	4000	500	500
	SDoH	3964	464	–
Text classification	Ade-corpus-v2 (*21*)	4000	500	500
	SemdClass (*26*)	2400	600	600
	DS	2127	237	–
Question answering	MedMCQA (*27*)	34,994	4183	4183
Natural language inference	BioNLI (*28*)	5544	6308	12,807

#### Three retrievers

The three retrievers—BM25, Contriever, and MedCPT—use a structured process to retrieve the most relevant instances for enhancing LLM predictions. This process consists of three main steps: (i) The retrievers first compute the vector representation of the given input sentence using their respective retrieval models. BM25 uses term-based weighting, while Contriever and MedCPT rely on dense embeddings generated through neural networks. (ii) Each retriever then calculates the vector representation for every instance in the retrieval corpus. BM25 relies on traditional lexical matching, while Contriever and MedCPT use deep learning–based embeddings to encode semantic information. (iii) Last, the retrievers measure the similarity between the input sentence vector and each instance vector in the corpus. The most relevant instances, determined by the highest similarity scores, are selected and fed into the LLM alongside the input sentence as examples, helping the model generate more accurate predictions.

#### Triple extraction dataset

In this paper, we used ADE, Chemprot, and GIT as the foundational datasets.

1. ADE is extended from the relation extraction task to the triplet extraction task in this paper. All sentences either describe the effect of the drug or the dose of the drug. Thus, the triplets consist of (head entity: drug, relation type: effect, tail entity: effect_description) and (head entity: drug, relation type: dosage, tail entity: dose_description). Among all triplets, there are only two relation types: effect and dosage.

2. ChemProt: The Chemical Protein Interaction Corpus comprises 2432 PubMed abstracts annotated with chemical-protein interactions, encompassing 23 distinct interaction relations. Building upon prior research (*19*), the corpus exclusively considers sentence-level instances, with a particular focus on five prominent interaction types for classification: CPR3, CPR4, CPR5, CPR6, and CPR9.

3. GIT is a high-quality biomedical triple extraction dataset for nondrug therapies, characterized by its high-quality annotations and comprehensive coverage of relation types. It includes 22 relation types from SemMedDB.

#### Link prediction

In this paper, we used PHarmKG and Hetionet as the foundational datasets in the link prediction task.

1. PHarmKG is a knowledge graph to describe the relationship among genes, drugs, and diseases. In this work, we aim to predict the four mentioned relation types (Interactions, Disease-Gene, Disease-Chemical, and Chemical-Gene) between two entities. During the huge quantity of triples in the PHarmKG, we randomly select 4000 samples from the source training set for training, 500 samples from the source testing set for testing, and 500 samples from the source validation set for validation.

2. Hetionet is an integrative network of disease, which includes 46 relation types. In our paper, we randomly select 4000 samples from the source training set for training, 500 samples from the source testing set for testing, and 500 samples from the source validation set for validation.

3. Dietary supplement (DS). This task determines whether a dietary supplement is positively or negatively associated with a specific event, or if there is no direct relationship. It facilitates the identification of cause-effect relationships or therapeutic uses of dietary supplements. For example, the task would identify a negative association between “ginseng” and “nausea.”

#### Text classification

In this paper, we used Ade-corpus-v2 and SemdClass as the foundational dataset in the text classification task.

1. The ADE-Corpus-V2 dataset is designed to classify whether a sentence is related to an adverse drug event (ADE) (true) or not (false). In our paper, we randomly select 4000 instances for training, 500 for testing, and 500 for validation.

2. The SemdClass aims to understand whether the provided triple belongs to the given sentence or not. It includes two classes, false and true.

3. SDoH focuses on classifying Social and Behavioral Determinants of Health snippets. The dataset consists of 2364 annotated notes, categorized into 20 broad SDoH categories, covering various social factors that influence health outcomes. These categories include employment status, work conditions, access to food, housing status, income status, health literacy, insurance status, access to transportation, maltreatment history, environmental conditions, housing quality, neighborhood security, crime/incarceration history, social discrimination, social isolation, social support, immigrant status, substance abuse, physical activity, and sleep quality.

#### QA and natural language inference

In this paper, we used MedMCQA as the foundational dataset in the QA task and used BioNLI as the dataset of natural language inference.

1. MedMCQA is a multiple-choice QA dataset that is designed to address the medical entrance exam questions. In this work, we opt for the five-choice version (A, B, C, D, and E).

2. BioNLI aims to understand whether the provided hypothesis is consistent or adversarial to the premise.

### Biomedical Retrieval-Augmented Generation Benchmark

In this part, we begin by outlining the operational flow of the RALs. Following this, we introduce the proposed four abilities and the building progress of four relevant testbeds. Last, we introduce the evaluation metrics used to assess performance.

#### RAL working flow

**Fig. 2.**
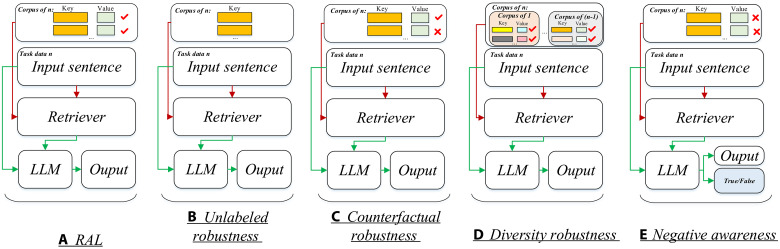
Overview of four testbeds on BioRAB. *n* refers to the special dataset for each task, such as Ade-corpus-v2 (text classification), and PHharmKG (link prediction). (**A**) represents the RL framework, where the retrieval corpus is labeled; (**B**) denotes the unlabeled retrieval corpus; and (**C**) refers to the retrieval corpus with counterfactual labels. In (**D**), the corpus of *n* refers to the set that includes the task datasets but excludes the training set of *n*. In (**E**), to distinguish the difference between “Output” and “True/False,” the “Output” is defined as the expected output for different tasks; for example, in the triple extraction task, the output is the triple. “True/False” refers to “the retrieved example is a negative example or the retrieved example is not a negative example.” In our work, the *n* corpus of *n* refers to the training set of *n*.

To solve the hallucination problem, RAL is proposed to retrieve the external knowledge from the corpus and improve the LLM performance. Generally, as shown in Fig. 2A, retrieved corpus needs to be constructed initially; in numerous QA RAL models, the corpus primarily originates from the unlabeled open source such as PubMed and Textbook. However, for some label-sensitive tasks, such as triple extraction, the unlabeled open source may be invalid. In our work, the corpus is defined as the training set for the relevant task. For instance, as illustrated in Fig. 2A, if “*n*” denotes the PHarmKG, each key corresponds to a sentence “what is the relationship between the head entity and tail entity?” in its training set, while the corresponding value denotes the relevant label *relationship* for that key. In the second step, the retriever is used to obtain the relevant (key, value) pairs from the corpus based on the input sentence. Last, the retrieved (key, value) pairs with the input sentence are fed into the LLM to generate the expected output. For each instance *X* of each *n*, there are three components: Instruction *I*, context *C*, and response *R*. For example, in the training dataset of Ade-corpus-v2 (classification task), if the label of the sentence S: “She had been administered tacrolimus for prophylaxis of graft-versus-host reaction” is False in the X, I = “You are an excellent linguist. The task is to predict whether this sentence is True or False. Examples: context: The hemangioma regressed markedly 6 weeks after the procedure and serous retinal detachment showed marked resolution response: False,” C = S, R = False.

#### Four abilities of BioRAL

Despite the fact that RAL has achieved considerable success in solving the hallucination problem, in the biomedical domain, the ability of RAL is underexplored. First, not all tasks have vast labeled corpora. While many research endeavors use the training set as the corpus, they still encounter limitations when contrasted with larger corpora. If a RAL can achieve similar performance to the RAL that uses labeled corpus, it would demonstrate the former’s ability to operate effectively without relying on labeled data. For another, the RAL may easily be misled by incorrectly labeled information (as shown in Fig. 2C). Furthermore, RALs may have the capability to obtain useful information from labeled corpora of other tasks (as shown in Fig. 2D). However, retrieving knowledge from labeled corpora of other tasks may introduce noise and potentially mislead the generation process. Last, when the retriever retrieves mislabeled (or counterfactual) information, the RAL may have the ability to discern that the retrieved knowledge is not conducive to output generation (as shown in Fig. 2E). To this end, we built the BioRAB to evaluate the ability of RAL in the biomedical domain, and we proposed four testbeds to test these abilities. In the next, we will detail these four abilities and how to construct the testbeds.

*Unlabeled robustness*. Not all tasks have vast labeled retrieval corpus; therefore, for each task, the retriever must gather information from unlabeled corpora, while the RAL may still have the ability to generate the expected results. To evaluate the efficacy of RAL in this regard, we introduce our proposed unlabeled robustness testbed. Specifically, as shown in Fig. 2B, the corpus of *n* is defined as the training set without value(label) for the *n*. The retriever retrieves the relevant information from this unlabeled corpus. After that, the retrieved Key with the input sentence is fed into the LLM. For example, in the training dataset of Ade-corpus-v2 (classification task), if the label of a sentence S: “She had been administered tacrolimus for prophylaxis of graft-versus-host reaction” is False in the X, I = “You are an excellent linguist. The task is to predict whether this sentence is True or False, retrieved sentence: A macrophage activation syndrome, possibly related to methotrexate toxicity, developed in a boy with systemic juvenile rheumatoid arthritis,” C = S, R = False.

*Counterfactual robustness*. Constructing a high-quality annotation corpus is challenging work, as it often involves dealing with incorrect data labeling. In our work, these mislabeled instances are called counterfactual instances. In the condition of the mislabeled corpus, the RAL may have the ability to avoid negative information. To validate the counterfactual robustness, we introduced our counterfactual robustness testbed. Specifically, as shown in Fig. 2C, when constructing the corpus of *n*, we set the negative rate to be 20, 80, or 100%, corresponding to 20, 80, or 100% of instances being wrongly labeled, respectively. An example of incorrect annotation in a classification dataset would be if there are two labels, “True” and “False.” If the true class of one instance is True, then its incorrect annotation would be False. Subsequently, the retriever is tasked with retrieving relevant information from this corpus. The retrieved information, along with the input sentence, is fed into the LLM to generate the output.

*Diverse robustness*. Diverse Robustness refers to the ability to incorporate diverse information from various task corpora. On the one hand, in numerous scenarios, the corpus from other tasks may contain valuable information to aid in generation. For instance, in the task of triple extraction, if a suitable triple extraction corpus is unavailable, the QA corpus may assist in extracting the necessary information. On the other hand, different tasks may introduce noise that could potentially impede the performance of the RAL. To generate better output, it is necessary for RAL to have the ability to retrieve diverse information. Thus, we introduce our diverse robustness testbed, as shown in Fig. 2D, when constructing the corpus of *n*, it incorporates corpora from other tasks. For instance, if *n* refers to the Chemprot (triple extraction task), the corpus of *n* includes corpora from tasks such as GIT (triple extraction task), PHarmKG (link prediction task), and so on. Next, the retriever is required to extract the pertinent information from the diverse corpus. Subsequently, the retrieved information, along with the input sentence, is fed into the LLM to generate the output.

*Negative awareness*. Negative awareness evaluates the ability of LLMs to discern whether the retrieved information is negative (it is not conducive to the expected output). In real-world scenarios, if the retriever obtains negative information and the LLM can identify it as such, the LLM can then seek out more useful information to aid in generation based on this feedback. Thus, we introduce our negative awareness testbed, as shown in Fig. 2E, and we designate all values in the corpus of *n* as incorrect labels. After obtaining the retrieved documents from the corpus, the model is expected to produce two types of output. First, in the task-based output, such as in the task of triple extraction, the output should be triple. Second, the model should also provide a judgment on whether the retrieved knowledge is negative or not.

#### Evaluation metrics

*Task-based metrics*. In the triple extraction task, same as BiomedRAG (*20*), triple is regarded as correct when its relation type, the head entity, and the tail entity are all correct. For example, in the sentence *Infusion of prostacyclin* (*PGI2*) *reportedly attenuates renal ischemic injury in the dog and the rat.*, triple *<Infusion*, *treats*, *rat>* is regarded as correct while *<injury*, *treats*, *rat>* is not. We evaluated all the models and reported the evaluation metric, including Micro Precision, Recall, and F1 score. For the text classification, link prediction, and QA task, we follow the same evaluation metrics as triple extraction. For the natural language inference task, we use the same evaluation metric (Macro F1) as the BioNLI.

*Negative awareness metrics.* To assess negative awareness in our study, we define a negative instance as a mislabeled instance. In the first, we need to evaluate the model performance using mislabeled examples. For instance, in the Ade-corpus-v2 classification data, with two labels True and False, this evaluation gauges the performance of True or False predictions.

Typically, in the RAL framework, if the retrieved example contains the input sentence and its expected output, the LLM should achieve 100% performance when tested with the input sentence. Despite all instances in the retrieval corpus being mislabeled, the LLM may still generate the correct output when using these examples. In our experiments, we also investigate this aspect. Building on this discovery, we delineate two types of negative instances:

1. True negatives: When the negative instance is provided to the LLM along with the input sentence, resulting in the incorrect output. In this scenario, the number of input sentences is denoted as *l^t^*.

2. False negatives: When the negative instance is presented to the LLM alongside the input sentence, leading to the correct output. In this case, the number of input sentences is represented as *l^f^*.

At the same time, we also expected the LLM could output “True—The retrieved example is negative example or False—The retrieved example is not a negative example” by providing a specific instruction, “Please determine whether the retrieved example constitutes negative information. If it is negative, please output False; if it is not negative, please output True” for each input sentence. For an input sentence that has false-negative examples, if the LLM could output “False—The retrieved example is not a negative example,” it demonstrates that the LLM recognizes the example as a false negative. After the judgment of LLM, the count of input sentences with “false negative examples” is denoted as f. For an input sentence that has true negative examples, if the LLM could output “True—The retrieved example is a negative example,” it demonstrates that the LLM recognizes the example as a true negative. After the judgment of LLM, the count of input sentences with “true negative examples” is denoted as *t*. Thus, the true negative awareness rate is calculated by *t/l^t^*, and the false negative awareness rate is calculated by *f/l^f^*.

### Improving the robustness and awareness of RALs

In this part, we mainly introduce our method to improve the robustness (counterfactual and unlabeled) and negative awareness ability of the RAL.

#### Improving the counterfactual robustness of RALs

In the context of mitigating mislabeled instances within a retrieved corpus, we propose a two-step approach consisting of a detection phase followed by a correction phase. This methodology ensures that errors introduced during data annotation or retrieval are systematically identified and rectified, thereby improving the quality of the corpus used for downstream tasks. In this work, we primarily focus on developing the testbed and therefore adopt a straightforward approach to address this issue. The investigation of more advanced methods is left for future work. More specifically, the first step in our approach involves developing a mislabeled instance detector that systematically identifies erroneous labels. Following the detection phase, the mislabeled instance corrector is responsible for modifying the identified erroneous labels. Specifically, for example, in the case of a text classification task, we leverage in-context learning (ICL) with LLMs (e.g., GPT-4) to assess the correctness of assigned labels, and use the internal knowledge to modify it. The prompt used for this process is as follows:

“This is a text classification task. Please determine whether the label assigned to the input sentence is correct. If the label is incorrect, please provide the correct label.”

Through this mechanism, the model systematically reviews each labeled instance and flags those suspected to be mislabeled. By using the insights from the LLM’s evaluation, the incorrect labels are revised accordingly.

#### Improving the unlabeled robustness of RALs

In the case of an unlabeled corpus, to enhance robustness in the absence of labeled data, we also leverage ICL with LLMs to assign labels. This is achieved by providing task-specific instructions. The detailed prompt is as follows (using text classification as an example):

“This is a text classification task aimed at determining whether a given sentence is related to an Adverse Drug Event (ADE). Please assign a label to each provided sentence to support this task.”

#### Improving the awareness of RALs

To enhance the negative awareness of RALs, a key direction is to improve the model’s ability to distinguish between positive instances in the corpus—which contribute to generating the correct answer—and negative instances, which do not.

For each input sentences x , there are hundreds or thousands of instance in the corpus not related to answer generation. On the basis of this observation, we design model *S* to help distinguish which instances are helpful for the final generation of the input x . This model takes the following information as input: (i) input sentence x ; (ii) positive instance $C^{p}=\left\{e^{p}\right\}$ , which is constructed based on the relevance of x ; in general, x itself serves as the most informative positive instance; (iii) negative entity set $C^{n}=\left\{e^{n}\right\}$ , which is created by randomly sampling the instances from the corpus that are not included in $C^{p}$ . To differentiate the positive and negative entities, *S* uses the LLM model f(.) , such as llama models to extract a semantic representation f(x) , $f\left(e^{p}\right)$ , and $f\left(e^{n}\right)$ for x , $e^{p}$ , and $e^{n}$ , respectively, and select the positive instances by measuring their distance to the input sentences. S is optimized with the following triple loss

$$\text{max}\left[∥f\left(x\right)-f\left(e^{p}\right)∥-∥f\left(x\right)-f\left(e^{n}\right)∥+\alpha ,0\right]$$

where ∥.∥ denotes the Euclidean distance and α is a margin parameter, which we set to 1 as default. During training, the triplet loss reduces the distance between f(x) and $f\left(e^{p}\right)$ while enlarging the distance between f(x) and $f\left(e^{n}\right)$ . Once the f(.) is trained, it is subsequently used for instruction tuning in the downstream task. For example, in the task SDoH classification, the trained f(.) based on Phi4 is used to fine-tune the model on the classification dataset.

## Supplementary Materials

### This PDF file includes:

Supplementary Text Tables S1 to S5 References
