# Supplementary material for: Benchmarking retrieval-augmented large language models in biomedical NLP: Application, robustness, and self-awareness
Source: Sci Adv. 2025 Nov 21;11(47):eadr1443. doi: 10.1126/sciadv.adr1443 (PMC12637297; doi:10.1126/sciadv.adr1443)
Supplement: Supplementary file 1 — Supplementary Text Tables S1 to S5 References [file sciadv.adr1443_sm.pdf]

Science Advances
AAAS

Supplementary Materials for

Benchmarking retrieval-augmented large language models in biomedical NLP: Application, robustness, and self-awareness

Mingchen Li *et al.*

Corresponding author: Rui Zhang, ruizhang@umn.edu

Sci. Adv. **11**, eadr1443 (2025)
DOI: 10.1126/sciadv.adr1443

This PDF file includes:

Supplementary Text
Tables S1 to S5
References

A Supplementary Materials

A.1 Paper structure summarization

Our paper primarily focuses on evaluating the effectiveness of current Retrieval-Augmented Learning (RAL) models in the biomedical domain. The paper is organized as follows: (1) we collect and analyze existing RAL models and compile 11 biomedical datasets spanning five different tasks; (2) we propose four testbeds to systematically evaluate these models (Section Introduction); (3) we introduce methods to address key challenges, such as enhancing robustness on unlabeled data and improving negative awareness (Section Materials and Methods); and (4) we present experimental results and discussions (Section Results and Section Discussion). Additionally, we provide a glossary table S1 and S2 summarizing the algorithms, evaluation metrics, references, descriptions, advantages, and limitations to enhance clarity and readability.

Task:	triple extraction, link prediction, text classification, question answering, and natural language inference
LLMs:	LLamA2-13B , MedLLamA-13B, LLaMA3 8B, Phi4 14B and Qwen2.5 32B
Retrievers:	BM25, Contriver, and MedCPT.
Dataset:	ADE, ChemProt, GIT, PHarmKG, Hetionet, Ade-corpus-v2, SemedCLass, MedMCQA, BioNLI, DS and SDoH.
Metrics:	Micro F1, Recall, Precision, Macro-F1,Negative Awareness Metrics
testbed:	Unlabeled Robustness, Counterfactual Robustness, Diverse Robustness, Negative Awarenes
Our methods:	Detect-and-Correct,contrastive learning approach

Table S1: Glossary of tasks, LLMs, retrivers, dataset, metrics, testbeds, our method

A.2 RESULTS OF RALS AND BACKBONE LLMS

Category	Name	Reference	Description	Advantages	Limitations
Retrieval Algorithms	BM25	(14)	Traditional term-based ranking model for information retrieval.	Fast and interpretable, effective for keyword-based search.	Limited to lexical overlap, does not capture semantics.
	Contriever	(15)	Dense retrieval model using contrastive learning for embeddings.	Captures semantic meaning, works well with LLMs.	Computationally expensive, requires pretraining.
	MedCPT	(16)	Medical domain-specific retrieval model combining contrastive pretraining and biomedical knowledge.	Tailored for biomedical data, improves retrieval quality.	Requires domain-specific data for training.
Evaluation Metrics	Micro Precision, Recall, F1	Standard ML Metric	Measures classification accuracy in terms of precision and recall.	Standard metric for classification tasks.	Does not account for retrieval-specific errors.
	Negative Awareness Rate	Proposed in this work	Measures how well the model detects false negatives and true negatives in retrieval.	Provides insight into retrieval robustness and bias.	May conflict with task-based metrics if miscalibrated.
Fine-Tuning & Loss Functions	Triplet Loss	(29)	Ensures that positive instances are closer in embedding space than negative instances.	Effective for contrastive learning and retrieval tasks.	Requires careful selection of triplets for training.
	Detect-and-Correct	This work	Identifies and corrects mislabeled instances to improve retrieval robustness.	Reduces the impact of counterfactuals and noisy retrieval.	Performance depends on retriever quality.

Table S2: Glossary of Algorithms and Metrics with References, Descriptions, Advantages, and Limitations.

Dataset	LLM	Approach	triple Precision	triple Recall	triple F1	head entity Precision	head entity Recall	head entity F1	relation Precision	relation Recall	relation F1	tail entity Precision	tail entity Recall	tail entity F1
ADE	LLaMA2-13B	BM25	30.99	30.88	30.93	73.96	73.71	73.84	94.85	94.54	94.70	49.10	48.94	49.02
		Contriever	36.07	36.06	36.06	79.76	79.72	79.74	93.80	93.76	93.78	48.99	48.97	48.98
		MedCPT	30.81	30.80	30.81	76.75	76.71	76.73	94.41	94.37	94.39	43.87	43.85	43.86
		No Retriever	34.94	34.79	34.86	83.64	83.29	83.46	95.29	94.88	95.08	42.10	41.92	42.01
	MedLLaMA-13B	BM25	33.77	33.76	33.77	77.06	77.02	77.04	94.82	94.77	94.80	51.20	51.18	51.19
		Contriever	35.66	33.57	34.58	79.15	74.51	76.76	94.36	88.83	91.51	49.18	46.29	47.69
		MedCPT	33.30	29.72	31.41	79.01	70.52	74.52	95.48	85.21	90.05	45.77	40.85	43.17
		No Retriever	12.26	12.16	12.21	81.87	81.22	81.55	95.69	94.93	95.31	15.66	15.54	15.60
	LLaMA3-8B	BM25	27.88	27.70	27.79	72.87	72.39	72.63	94.61	93.99	94.30	45.79	45.49	45.64
		Contriever	34.44	31.13	32.70	78.91	71.31	74.92	93.14	84.18	88.43	48.57	43.90	46.12
		MedCPT	31.70	27.04	29.19	77.99	66.53	71.80	94.00	80.19	86.55	45.35	38.69	41.75
		No Retriever	9.85	5.49	7.05	83.84	46.76	60.04	96.30	53.71	68.96	13.05	7.28	9.34
	Phi4 14B	BM25	37.63	34.93	36.23	79.16	73.47	76.21	94.69	87.89	91.16	54.43	50.52	52.40
		Contriever	31.14	24.88	27.66	77.67	62.07	69.00	93.71	74.88	83.25	45.83	36.62	40.71
		MedCPT	28.82	24.27	26.35	79.77	67.18	72.94	93.98	79.15	85.93	41.64	35.07	38.07
		No Retriever	16.73	16.38	16.56	81.02	79.34	80.17	95.54	93.57	94.54	21.62	21.17	21.39
	Qwen2.5 32B	BM25	40.79	40.33	40.56	80.34	79.44	79.89	94.87	93.80	94.33	56.60	55.96	56.28
		Contriever	46.24	45.07	45.65	81.89	79.81	80.84	94.27	91.88	93.06	57.47	56.01	56.73
		MedCPT	44.18	41.17	42.62	82.02	76.43	79.13	94.96	88.50	91.62	55.72	51.92	53.75
		No Retriever	31.19	23.43	26.76	84.75	63.66	72.71	96.75	72.68	83.00	36.31	27.28	31.15
ChemProt	LLaMA2-13B	BM25	49.44	48.78	49.11	65.77	64.89	65.33	75.56	74.55	75.05	65.51	64.63	65.07
		Contriever	85.75	85.05	85.40	98.42	97.61	98.01	91.58	90.84	91.21	94.02	93.25	93.63
		MedCPT	86.25	85.40	85.82	98.44	97.47	97.95	91.19	90.29	90.73	95.53	94.59	95.06
		No Retriever	78.58	76.41	77.48	98.12	95.40	96.74	90.70	88.19	89.43	88.21	85.78	86.98
	MedLLaMA-13B	BM25	54.78	49.53	52.02	73.30	66.29	69.62	78.10	70.62	74.17	74.40	67.28	70.66
		Contriever	86.15	85.22	85.69	97.97	96.92	97.44	91.38	90.40	90.89	95.18	94.15	94.66
		MedCPT	81.33	80.08	80.70	98.14	96.63	97.38	89.60	88.22	88.91	91.96	90.55	91.25
		No Retriever	52.10	49.04	50.52	97.65	91.91	94.69	90.82	85.48	88.07	57.91	54.50	56.15
	LLaMA3-8B	BM25	70.23	69.98	70.10	83.86	83.57	83.71	84.30	84.00	84.15	91.54	91.22	91.38
		Contriever	87.13	86.68	86.91	97.92	97.41	97.67	91.26	90.78	91.02	95.56	95.06	95.30
		MedCPT	86.62	83.07	84.81	98.06	94.04	96.01	92.54	88.74	90.60	94.06	90.20	92.09
		No Retriever	23.21	19.72	21.32	98.63	83.80	90.61	91.75	77.95	84.29	26.09	22.16	23.97
	Phi4 14B	BM25	82.46	80.10	81.26	95.09	92.38	93.71	90.42	87.84	89.11	91.95	89.33	90.62
		Contriever	71.36	68.12	69.70	98.60	94.12	96.31	93.39	89.15	91.22	76.66	73.18	74.88
		MedCPT	81.48	77.57	79.48	97.71	93.02	95.31	92.09	87.67	89.82	88.45	84.21	86.28
		No Retriever	79.09	75.45	77.23	99.02	94.47	96.70	93.38	89.09	91.19	85.18	81.27	83.18
	Qwen2.5 32B	BM25	86.46	86.01	86.24	98.27	97.76	98.02	90.18	89.70	89.94	95.94	95.43	95.68
		Contriever	87.26	87.06	87.16	98.34	98.11	98.22	90.06	89.85	89.95	97.20	96.97	97.09
		MedCPT	87.86	87.58	87.72	98.51	98.20	98.35	90.90	90.61	90.75	96.38	96.07	96.23
		No Retriever	88.38	87.81	88.09	98.68	98.05	98.37	91.92	91.33	91.63	95.49	94.88	95.19
GIT	LLaMA2-13B	BM25	60.81	54.73	57.61	76.94	69.25	72.89	76.70	69.03	72.67	77.78	70.00	73.68
		Contriever	74.78	72.37	73.55	89.44	86.56	87.98	83.22	80.54	81.86	87.11	84.30	85.68
		MedCPT	75.64	73.44	74.52	92.03	89.35	90.67	83.06	80.65	81.83	89.04	86.45	87.73
		No Retriever	61.76	56.45	58.99	84.12	76.88	80.34	73.53	67.20	70.22	82.94	75.81	79.21
	MedLLaMA-13B	BM25	58.59	57.20	57.89	79.30	77.42	78.35	72.91	71.18	72.03	78.63	76.77	77.69
		Contriever	65.95	65.81	65.88	85.34	85.16	85.25	75.86	75.70	75.78	83.84	83.66	83.75
		MedCPT	75.65	74.84	75.24	90.22	89.25	89.73	82.39	81.51	81.95	90.00	89.03	89.51
		No Retriever	42.60	41.51	42.05	89.18	86.88	88.02	75.28	73.33	74.29	52.76	51.40	52.07
	LLaMA3-8B	BM25	62.72	62.58	62.65	79.31	79.14	79.22	79.74	79.57	79.66	77.80	77.63	77.72
		Contriever	74.76	67.53	70.96	89.76	81.08	85.20	82.14	74.19	77.97	88.33	79.78	83.84
		MedCPT	66.20	50.97	57.59	87.15	67.10	75.82	82.96	63.87	72.17	78.77	60.65	68.53
		No Retriever	75.81	72.80	74.27	87.79	84.30	86.01	85.55	82.15	83.82	86.67	83.23	84.91
	Phi4 14B	BM25	64.18	57.42	60.61	84.62	75.70	79.91	78.37	70.11	74.01	77.16	69.03	72.87
		Contriever	75.49	74.19	74.84	89.50	87.96	88.72	83.37	81.94	82.65	87.75	86.24	86.98
		MedCPT	73.51	71.61	72.55	88.30	86.02	87.15	81.68	79.57	80.61	87.64	85.38	86.49
		No Retriever	30.28	29.89	30.09	87.15	86.02	86.58	76.69	75.70	76.19	41.83	41.29	41.56
	Qwen2.5 32B	BM25	66.19	64.84	65.51	85.62	83.87	84.74	78.49	76.88	77.68	78.59	76.99	77.78
		Contriever	73.12	73.12	73.12	87.20	87.20	87.20	81.29	81.29	81.29	85.70	85.70	85.70
		MedCPT	69.46	69.46	69.46	86.24	86.24	86.24	79.35	79.35	79.35	84.95	84.95	84.95
		No Retriever	68.72	68.28	68.50	86.36	85.81	86.08	78.79	78.28	78.53	84.31	83.76	84.03

Table S3: Results of various approaches for triple extraction on ADE, ChemProt, and GIT. Underline with shade (green, pink, and blue) indicates the best performance on ADE, ChemProt, and GIT separately.

		Link Prediction						Text Classification						Question Answering			Natural Language Inference
		PHarmKG			Hetionet			Ade-corpus-v2			SemClass			MedMCQA			BioNLI
LLM	Approach	Precision	Recall	F1	Precision	Recall	F1	Precision	Recall	F1	Precision	Recall	F1	Precision	Recall	F1	Macro-avg F1
LLaMA2-13B	BM25	97.60	97.60	97.60	82.37	82.37	82.37	95.40	95.40	95.40	75.50	75.50	75.50	40.38	40.49	40.42	45.10
	Contriever	98.00	98.00	98.00	77.00	77.00	77.00	96.60	96.60	96.60	79.33	79.33	79.33	35.53	35.52	35.52	35.12
	MedCPT	97.40	97.40	97.40	81.60	81.60	81.60	96.80	96.80	96.80	78.50	78.50	78.50	36.78	36.93	36.80	69.21
	No Retriever	97.60	97.60	97.60	80.80	80.80	80.80	96.40	96.40	96.40	77.66	77.66	77.66	41.63	41.52	41.52	62.62
MedLLaMA-13B	BM25	95.00	95.00	95.00	90.40	90.40	90.40	95.60	95.60	95.60	72.67	72.67	72.67	37.81	37.96	37.86	48.81
	Contriever	97.00	97.00	97.00	77.20	77.20	77.20	95.60	95.60	95.60	77.66	77.66	77.66	29.82	29.75	29.77	53.07
	MedCPT	97.40	97.40	97.40	84.40	84.40	84.40	95.40	95.40	95.40	76.16	76.16	76.16	33.86	34.04	33.88	53.68
	No Retriever	97.20	97.20	97.20	78.54	78.54	78.54	95.40	95.40	95.40	64.00	64.00	64.00	46.79	46.41	46.47	61.07
LLaMA3-8B	BM25	96.80	96.80	96.80	81.80	81.80	81.80	94.80	94.80	94.80	75.50	75.50	75.50	37.73	38.90	37.79	19.17
	Contriever	96.60	96.60	96.60	73.40	73.40	73.40	94.60	94.60	94.60	75.83	75.83	75.83	28.11	28.12	28.11	63.85
	MedCPT	97.00	97.00	97.00	83.00	83.00	83.00	95.40	95.40	95.40	74.67	74.67	74.67	31.57	31.82	31.56	56.89
	No Retriever	97.20	97.20	97.20	81.80	81.80	81.80	93.80	93.80	93.80	73.16	73.16	73.16	56.93	55.43	55.91	6.71
Phi4 14B	BM25	97.20	97.20	97.20	88.80	88.80	88.80	88.20	88.20	88.20	74.67	74.67	74.67	39.33	39.62	39.42	67.65
	Contriever	31.60	31.60	31.60	74.20	74.20	74.20	90.80	90.80	90.80	75.17	75.17	75.17	35.38	34.81	33.89	56.65
	MedCPT	96.80	96.80	96.80	82.80	82.80	82.80	90.60	90.60	90.60	75.00	75.00	75.00	29.72	26.74	27.72	85.45
	No Retriever	97.60	97.60	97.60	82.20	82.20	82.20	89.20	89.20	89.20	72.50	72.50	72.50	66.46	66.01	66.18	82.50
Qwen2.5 32B	BM25	97.20	97.20	97.20	82.00	82.00	82.00	94.20	94.20	94.20	78.50	78.50	78.50	29.67	27.41	28.39	81.29
	Contriever	97.60	97.60	97.60	75.80	75.80	75.80	94.20	94.20	94.20	76.33	76.33	76.33	28.96	27.44	28.10	89.99
	MedCPT	96.20	96.20	96.20	82.80	82.80	82.80	95.00	95.00	95.00	76.83	76.83	76.83	29.15	27.53	28.22	91.19
	No Retriever	90.40	90.40	90.40	81.00	81.00	81.00	94.80	94.80	94.80	48.00	48.00	48.00	24.69	18.30	20.91	63.20

Table S4: Results of various approaches for link prediction, text classification, question answering, and natural language inference. Underline with green shade indicates the best performance on each dataset.

LLM	Approach	DS link prediction			SDH classification		
		Precision	Recall	F1	Precision	Recall	F1
LLaMA2-13B	BM25	75.86	75.86	75.86	63.71	63.71	63.71
	Contriever	76.50	76.50	76.50	61.18	61.18	61.18
	MedCPT	78.01	78.01	78.01	68.77	68.77	68.77
	No Retriever	77.15	77.15	77.15	72.57	72.57	72.57
MedLLaMA-13B	BM25	71.33	71.33	71.33	65.40	65.40	65.40
	Contriever	75.64	75.64	75.64	56.96	56.96	56.96
	MedCPT	72.84	72.84	72.84	66.66	66.66	66.66
	No Retriever	80.38	80.38	80.38	67.71	67.71	67.71
LLaMA3-8B	BM25	75.43	75.43	75.43	71.30	71.30	71.30
	Contriever	78.01	78.01	78.01	72.57	72.57	72.57
	MedCPT	76.29	76.29	76.29	71.72	71.72	71.72
	No Retriever	80.38	80.38	80.38	57.00	57.00	57.00
Phi4 14B	BM25	60.75	60.75	60.75	78.90	78.90	78.90
	Contriever	53.58	53.58	53.58	81.43	81.43	81.43
	MedCPT	57.38	57.38	57.38	80.16	80.16	80.16
	No Retriever	54.05	54.05	54.05	74.68	74.68	74.68
Qwen2.5 32B	BM25	72.44	72.44	72.44	78.05	78.05	78.05
	Contriever	77.37	77.37	77.37	80.16	80.16	80.16
	MedCPT	71.78	71.78	71.78	72.99	72.99	72.99
	No Retriever	76.93	76.93	76.93	74.26	74.26	74.26

Table S5: Results of various approaches for two private datasets DS and SDH.

REFERENCES AND NOTES

1. C. Wu, W. Lin, X. Zhang, Y. Zhang, W. Xie, Y. Wang, PMC-LLaMA: Toward building open-source language models for medicine. *J. Am. Med. Inform. Assoc.* **31**, 1833–1843 (2024).

2. K. Singhal, T. Tu, J. Gottweis, R. Sayres, E. Wulczyn, M. Amin, L. Hou, K. Clark, S. R. Pfohl, H. Cole-Lewis, D. Neal, Q. M. Rashid, M. Schaekermann, A. Wang, D. Dash, J. H. Chen, N. H. Shah, S. Lachgar, P. A. Mansfield, S. Prakash, B. Green, E. Dominowska, B. A. y Arcas, N. Tomašev, Y. Liu, R. Wong, C. Semturs, S. S. Mahdavi, J. K. Barral, D. R. Webster, G. S. Corrado, Y. Matias, S. Azizi, A. Karthikesalingam, V. Natarajan, Toward expert-level medical question answering with large language models. *Nat. Med.* **31**, 943–950 (2025).

3. M. Li, H. Zhou, H. Yang, R. Zhang, RT: A retrieving and chain-of-thought framework for few-shot medical named entity recognition. *J. Am. Med. Inform. Assoc.* **31**, 1929–1938 (2024).

4. Z. Ji, N. Lee, R. Frieske, T. Yu, D. Su, Y. Xu, E. Ishii, Y. J. Bang, A. Madotto, P. Fung, Survey of hallucination in natural language generation. *ACM Comput. Surv.* **55**, 1–38 (2023).

5. O. Ovadia, M. Brief, M. Mishaeli, O. Elisha, Fine-tuning or retrieval? Comparing knowledge injection in LLMs. arXiv:2312.05934 [cs.AI] (2023).

6. P. Lewis, E. Perez, A. Piktus, F. Petroni, V. Karpukhin, N. Goyal, H. Küttler, M. Lewis, W. Yih, T. Rocktäschel, S. Riedel, D. Kiela, Retrieval-augmented generation for knowledge-intensive NLP tasks. *Adv. Neural Inf. Process. Syst.* **33**, 9459–9474 (2020).

7. M. Li, L. Huang, Understand the dynamic world: An end-to-end knowledge informed framework for open domain entity state tracking, in *Proceedings of the 46th International ACM SIGIR Conference on Research and Development in Information Retrieval (SIGIR), Association for Computing Machinery (ACM)* (2023), pp. 842–851.

8. J. Huang, M. Li, Z. Yao, Z. Yang, Y. Xiao, F. Ouyang, X. Li, S. Han, H. Yu, RiTeK: A dataset for large language models complex reasoning over textual knowledge graphs. arXiv:2410.13987 [cs.CL] (2024).

9. C. Zakka, R. Shad, A. Chaurasia, A. R. Dalal, J. L. Kim, M. Moor, R. Fong, C. Phillips, K. Alexander, E. Ashley, J. Boyd, K. Boyd, K. Hirsch, C. Langlotz, R. Lee, J. Melia, J. Nelson, K. Sallam, S. Tullis, M. A. Vogelsong, J. P. Cunningham, W. Hiesinger, Almanac–retrieval-augmented language models for clinical medicine. *NEJM AI* **1**, AIoa2300068 (2024).

10. H. Touvron, T. Lavril, G. Izacard, X. Martinet, M.-A. Lachaux, T. Lacroix, B. Rozière, N. Goyal, E. Hambro, F. Azhar, A. Rodriguez, A. Joulin, É. Grave, G. Lample, LLaMA: Open and efficient foundation language models. arXiv:2302.13971 [cs.CL] (2023).

11. A. Grattafiori, A. Dubey, A. Jauhri, A. Pandey, A. Kadian, A. Al-Dahle, A. Letman, A. Mathur, A. Schelten, A. Vaughan, A. Yang, A. Fan, A. Goyal, A. Hartshorn, A. Yang, A. Mitra, A. Sravankumar, A. Korenev, A. Hinsvark, A. Rao, A. Zhang, A. Rodriguez, A. Gregerson, A. Spataru, B. Roziere, B. Biron, B. Tang, B. Chern, C. Caucheteux, C. Nayak, C. Bi, C. Marra, C. M. Connell, C. Keller, C. Touret, C. Wu, C. Wong, C. C. Ferrer, C. Nikolaidis, D. Allonsius, D. Song, D. Pintz, D. Livshits, D. Wyatt, D. Esiobu, D. Choudhary, D. Mahajan, D. Garcia-Olano, D. Perino, D. Hupkes, E. Lakomkin, E. A. Badawy, E. Lobanova, E. Dinan, E. M. Smith, F. Radenovic, F. Guzmán, F. Zhang, G. Synnaeve, G. Lee, G. L. Anderson, G. Thattai, G. Nail, G. Mialon, G. Pang, G. Cucurell, H. Nguyen, H. Korevaar, H. Xu, H. Touvron, I. Zarov, I. A. Ibarra, I. Kloumann, I. Misra, I. Evtimov, J. Zhang, J. Copet, J. Lee, J. Geffert, J. Vranes, J. Park, J. Mahadeokar, J. Shah, J. van der Linde, J. Billock, J. Hong, J. Lee, J. Fu, J. Chi, J. Huang, J. Liu, J. Wang, J. Yu, J. Bitton, J. Spisak, J. Park, J. Rocca, J. Johnstun, J. Saxe, J. Jia, K. V. Alwala, K. Prasad, K. Upasani, K. Plawiak, K. Li, K. Heafield, K. Stone, K. El-Arini, K. Iyer, K. Malik, K. Chiu, K. Bhalla, K. Lakhotia, L. Rantala-Yeary, L. van der Maaten, L. Chen, L. Tan, L. Jenkins, L. Martin, L. Madaan, L. Malo, L. Blecher, L. Landzaat, L. de Oliveira, M. Muzzi, M. Pasupuleti, M. Singh, M. Paluri, M. Kardas, M. Tsimpoukelli, M. Oldham, M. Rita, M. Pavlova, M. Kambadur, M. Lewis, M. Si, M. K. Singh, M. Hassan, N. Goyal, N. Torabi, N. Bashlykov, N. Bogoychev, N. Chatterji, N. Zhang, O. Duchenne, O. Çelebi, P. Alrassy, P. Zhang, P. Li, P. Vasic, P. Weng, P. Bhargava, P. Dubal, P. Krishnan, P. S. Koura, P. Xu, Q. He, Q. Dong, R. Srinivasan, R. Ganapathy, R. Calderer, R. S. Cabral, R. Stojnic, R. Raileanu, R. Maheswari, R. Girdhar, R. Patel, R. Sauvestre, R. Polidoro, R. Sumbaly, R. Taylor, R. Silva, R. Hou, R. Wang, S. Hosseini, S. Chennabasappa, S. Singh, S. Bell, S. S. Kim, S. Edunov, S. Nie, S. Narang, S. Raparthy, S. Shen, S. Wan, S. Bhosale, S. Zhang, S. Vandenhende, S. Batra, S. Whitman, S.

Sootla, S. Collot, S. Gururangan, S. Borodinsky, T. Herman, T. Fowler, T. Sheasha, T. Georgiou, T. Scialom, T. Speckbacher, T. Mihaylov, T. Xiao, U. Karn, V. Goswami, V. Gupta, V. Ramanathan, V. Kerkez, V. Gonguet, V. Do, V. Vogeti, V. Albiero, V. Petrovic, W. Chu, W. Xiong, W. Fu, W. Meers, X. Martinet, X. Wang, X. Wang, X. E. Tan, X. Xia, X. Xie, X. Jia, X. Wang, Y. Goldschlag, Y. Gaur, Y. Babaei, Y. Wen, Y. Song, Y. Zhang, Y. Li, Y. Mao, Z. D. Coudert, Z. Yan, Z. Chen, Z. Papakipos, A. Singh, A. Srivastava, A. Jain, A. Kelsey, A. Shajnfeld, A. Gangidi, A. Victoria, A. Goldstand, A. Menon, A. Sharma, A. Boesenberg, A. Baevski, A. Feinstein, A. Kallet, A. Sangani, A. Teo, A. Yunus, A. Lupu, A. Alvarado, A. Caples, A. Gu, A. Ho, A. Poulton, A. Ryan, A. Ramchandani, A. Dong, A. Franco, A. Goyal, A. Saraf, A. Chowdhury, A. Gabriel, A. Bharambe, A. Eisenman, A. Yazdan, B. James, B. Maurer, B. Leonhardi, B. Huang, B. Loyd, B. De Paola, B. Paranjape, B. Liu, B. Wu, B. Ni, B. Hancock, B. Wasti, B. Spence, B. Stojkovic, B. Gamido, B. Montalvo, C. Parker, C. Burton, C. Mejia, C. Liu, C. Wang, C. Kim, C. Zhou, C. Hu, C.-H. Chu, C. Cai, C. Tindal, C. Feichtenhofer, C. Gao, D. Civin, D. Beaty, D. Kreymer, D. Li, D. Adkins, D. Xu, D. Testuggine, D. David, D. Parikh, D. Liskovich, D. Foss, D. Wang, D. Le, D. Holland, E. Dowling, E. Jamil, E. Montgomery, E. Presani, E. Hahn, E. Wood, E.-T. Le, E. Brinkman, E. Arcaute, E. Dunbar, E. Smothers, F. Sun, F. Kreuk, F. Tian, F. Kokkinos, F. Ozgenel, F. Caggioni, F. Kanayet, F. Seide, G. M. Florez, G. Schwarz, G. Badeer, G. Swee, G. Halpern, G. Herman, G. Sizov, Guangyi (Jack)Zhang, G. Lakshminarayanan, H. Inan, H. Shojanazeri, H. Zou, H. Wang, H. Zha, H. Habeeb, H. Rudolph, H. Suk, H. Aspegren, H. Goldman, H. Zhan, I. Damlaj, I. Molybog, I. Tufanov, I. Leontiadis, I.-E. Veliche, I. Gat, J. Weissman, J. Geboski, J. Kohli, J. Lam, J. Asher, J.-B. Gaya, J. Marcus, J. Tang, J. Chan, J. Zhen, J. Reizenstein, J. Teboul, J. Zhong, J. Jin, J. Yang, J. Cummings, J. Carvill, J. Shepard, J. M. Phie, J. Torres, J. Ginsburg, J. Wang, K. Wu, Kam Hou U, K. Saxena, K. Khandelwal, K. Zand, K. Matosich, K. Veeraraghavan, K. Michelena, K. Li, K. Jagadeesh, K. Huang, K. Chawla, K. Huang, L. Chen, L. Garg, Lavender A, L. Silva, L. Bell, L. Zhang, L. Guo, L. Yu, L. Moshkovich, L. Wehrstedt, M. Khabsa, M. Avalani, M. Bhatt, M. Mankus, M. Hasson, M. Lennie, M. Reso, M. Groshev, M. Naumov, M. Lathi, M. Keneally, M. Liu, M. L. Seltzer, M. Valko, M. Restrepo, M. Patel, M. Vyatskov, M. Samvelyan, M. Clark, M. Macey, M. Wang, M. J. Hermoso, M. Metanat, M. Rastegari, M. Bansal, N. Santhanam, N. Parks, N. White, N. Bawa, N. Singhal, N. Egebo, N. Usunier, N. Mehta, N. P. Laptev, N. Dong, N. Cheng, O. Chernoguz, O. Hart, O. Salpekar, O. Kalinli, P. Kent, P. Parekh, P. Saab, P. Balaji, P. Rittner,

P. Bontrager, P. Roux, P. Dollar, P. Zvyagina, P. Ratanchandani, P. Yuvraj, Q. Liang, R. Alao, R. Rodriguez, R. Ayub, R. Murthy, R. Nayani, R. Mitra, R. Parthasarathy, R. Li, R. Hogan, R. Battey, R. Wang, R. Howes, R. Rinott, S. Mehta, S. Siby, S. J. Bondu, S. Datta, S. Chugh, S. Hunt, S. Dhillon, S. Sidorov, S. Pan, S. Mahajan, S. Verma, S. Yamamoto, S. Ramaswamy, S. Lindsay, S. Lindsay, S. Feng, S. Lin, S. C. Zha, S. Patil, S. Shankar, S. Zhang, S. Zhang, S. Wang, S. Agarwal, S. Sajuyigbe, S. Chintala, S. Max, S. Chen, S. Kehoe, S. Satterfield, S. Govindaprasad, S. Gupta, S. Deng, S. Cho, S. Virk, S. Subramanian, S. Choudhury, S. Goldman, T. Remez, T. Glaser, T. Best, T. Koehler, T. Robinson, T. Li, T. Zhang, T. Matthews, T. Chou, T. Shaked, V. Vontimitta, V. Ajayi, V. Montanez, V. Mohan, V. S. Kumar, V. Mangla, V. Ionescu, V. Poenaru, V. T. Mihailescu, V. Ivanov, W. Li, W. Wang, W. Jiang, W. Bouaziz, W. Constable, X. Tang, X. Wu, X. Wang, X. Wu, X. Gao, Y. Kleinman, Y. Chen, Y. Hu, Y. Jia, Y. Qi, Y. Li, Y. Zhang, Y. Zhang, Y. Adi, Y. Nam, Yu (Sid)Wang, Y. Zhao, Y. Hao, Y. Qian, Y. Li, Y. He, Z. Rait, Z. De Vito, Z. Rosnbrick, Z. Wen, Z. Yang, Z. Zhao, Z. Ma, The LLaMA 3 herd of models. arXiv:2407.21783 [cs.AI] (2024).

12. M. Abdin, J. Aneja, H. Behl, S. Bubeck, R. Eldan, S. Gunasekar, M. Harrison, R. J. Hewett, M. Javaheripi, P. Kauffmann, J. R. Lee, Y. T. Lee, Y. Li, W. Liu, C. C. T. Mendes, A. Nguyen, E. Price, G. de Rosa, O. Saarikivi, A. Salim, S. Shah, X. Wang, R. Ward, Y. Wu, D. Yu, C. Zhang, Y. Zhang, Phi-4 technical report. arXiv:2412.08905 [cs.CL] (2024).

13. A. Yang, B. Yang, B. Zhang, B. Hui, B. Zheng, B. Yu, C. Li, D. Liu, F. Huang, H. Wei, H. Lin, J. Yang, J. Tu, J. Zhang, J. Yang, J. Yang, J. Zhou, J. Lin, K. Dang, K. Lu, K. Bao, K. Yang, L. Yu, M. Li, M. Xue, P. Zhang, Q. Zhu, R. Men, R. Lin, T. Li, T. Tang, T. Xia, X. Ren, X. Ren, Y. Fan, Y. Su, Y. Zhang, Y. Wan, Y. Liu, Z. Cui, Z. Zhang, Z. Qiu, Qwen2.5 technical report. arXiv:2412.15115 [cs.CL] (2024).

14. S. E. Robertson, K. Spärck Jones, Relevance weighting of search terms. *J. Am. Soc. Inf. Sci.* **27**, 129–146 (1976).

15. G. Izacard, M. Caron, L. Hosseini, S. Riedel, P. Bojanowski, A. Joulin, É. Grave, Unsupervised dense information retrieval with contrastive learning. arXiv:2112.09118 [cs. IR] (2021).

16. Q. Jin, W. Kim, Q. Chen, D. C. Comeau, L. Yeganova, W. J. Wilbur, Z. Lu, MedCPT: Contrastive pre-trained transformers with large-scale PubMed search logs for zero-shot biomedical information retrieval. *Bioinformatics* **39**, btad651 (2023).

17. G. Xiong, Q. Jin, Z. Lu, A. Zhang, "Benchmarking retrieval-augmented generation for medicine," in *Findings of the Association for Computational Linguistics: ACL 2024* (Association for Computational Linguistics, 2024), pp. 6233–6251.

18. E. W. Sayers, J. Beck, K. Brister, E. Bolton, S. Canese, D. Comeau, R. Funk, K. Kim, J. Kim, W. Klimke, W. Lee, J. Lu, T. Madden, A. Marchler-Bauer, V. Ostell, B. Phan, S. Rangwala, L. Schneider, F. Wang, D. Ye, Y. Zhang, Z. Zhao, PubMed: The NCBI database of biomedical literature. *Nucleic Acids Res.* **49**, D1388–D1395 (2021).

19. C. Sun, Z. Yang, L. Wang, Y. Zhang, H. Lin, J. Wang, MRC4BioER: Joint extraction of biomedical entities and relations in the machine reading comprehension framework. *J. Biomed. Inform.* **125**, 103956 (2022).

20. M. Li, H. Kilicoglu, H. Xu, R. Zhang, BiomedRAG: A retrieval-augmented large language model for biomedicine. *J. Biomed. Inform.* **162**, 104–769 (2025).

21. H. Gurulingappa, A. M. Rajput, A. Roberts, J. Fluck, M. Hofmann-Apitius, L. Toldo, Development of a benchmark corpus to support the automatic extraction of drug-related adverse effects from medical case reports. *J. Biomed. Inform.* **45**, 885–892 (2012).

22. O. Taboureau, S. K. Nielsen, K. Audouze, N. Weinhold, D. Edsgård, F. S. Roque, I. Kouskoumvekaki, A. Bora, R. Curpan, T. S. Jensen, S. Brunak, T. I. Oprea, ChemProt: A disease chemical biology database. *Nucleic Acids Res.* **39**, D367–D372 (2010).

23. M. Li, H. Zhou, R. Zhang, Benchmarking large language models in biomedical triple extraction. arXiv:2310.18463 [cs.CL] (2023).

24. S. Zheng, J. Rao, Y. Song, J. Zhang, X. Xiao, E. F. Fang, Y. Yang, Z. Niu, PharmKG: A dedicated knowledge graph benchmark for biomedical data mining. *Brief. Bioinform.* **22**, bbaa344 (2021).

25. D. S. Himmelstein, A. Lizee, C. Hessler, L. Brueggeman, S. L. Chen, D. Hadley, A. Green, P. Khankhanian, S. E. Baranzini, Systematic integration of biomedical knowledge prioritizes drugs for repurposing. *eLife* **6**, e26726 (2017).

26. J. A. Vasilakes, R. Rizvi, R. Zhang, Annotated Semantic Predications from SemMedDB (University of Minnesota Digital Conservancy, 2018); https://conservancy.umn.edu/handle/11299/194965.

27. A. Pal, L. K. Umapathi, M. Sankarasubbu, MedMCQA: A large-scale multi-subject multi-choice dataset for medical domain question answering, *Proceedings of Machine Learning Research (PMLR)* (2022), pp. 248–260.

28. M. Bastan, M. Surdeanu, N. Balasubramanian, BioNLI: Generating a biomedical NLI dataset using lexico-semantic constraints for adversarial examples, in *Findings of the Association for Computational Linguistics: EMNLP 2022, Association for Computational Linguistics (ACL)* (2022), pp. 5093–5104.

29. F. Schroff, D. Kalenichenko, J. Philbin, "FaceNet: A unified embedding for face recognition and clustering," in *Proceedings of the IEEE Conference on Computer Vision and Pattern Recognition* (*CVPR*) (IEEE, 2015), pp. 815–823.
